# Clinical Effectiveness of Roflumilast in Patients With Chronic Obstructive Pulmonary Disease: A Systematic Review and Meta-Analysis

**DOI:** 10.7759/cureus.114644

**Published:** 2026-08-17

**Authors:** Nithin Sai Yenugu, Hamza Bin Ashraf, Disha Disha, Shramik Rawal, Heer Joshi, Sonalben Chaudhary, Calvin R Wei, Shamsha Hirani

**Affiliations:** 1 Internal Medicine, HCA Houston Healthcare Clear Lake, Webster, USA; 2 Internal Medicine, Central Park Medical College, Lahore, PAK; 3 Medicine, St. Martinus University, Willemstad, CUW; 4 Internal Medicine, Mater Dei Hospital, Msida, MLT; 5 Internal Medicine, American University of Barbados, Bridgetown, BRB; 6 Internal Medicine, Zydus Sitapur Hospital, Sitapur, IND; 7 Research and Development, Shing Huei Group, Taipei, TWN; 8 Cardiology, Baqai Hospital, Karachi, PAK

**Keywords:** chronic obstructive pulmonary disease, copd, exacerbation, meta-analysis, phosphodiesterase-4 inhibitor, roflumilast

## Abstract

Chronic obstructive pulmonary disease (COPD) is a major contributor to global morbidity and mortality, with acute exacerbations accelerating disease progression and substantially increasing healthcare utilization and costs. To evaluate the available evidence on roflumilast, an oral phosphodiesterase-4 inhibitor, we performed a systematic review and meta-analysis. We searched PubMed/MEDLINE, Embase, CENTRAL, Web of Science, Scopus, and ClinicalTrials.gov from 1 January 2011 to 15 July 2026 for studies evaluating roflumilast in adults with COPD. Ten studies met the inclusion criteria, comprising randomized controlled trials, a quasi-experimental trial, and an observational cohort study. Roflumilast was associated with statistically significant improvements in forced expiratory volume in one second (FEV1) compared with control (pooled mean difference: 0.06 L, 95% CI: 0.04-0.08; I² = 72%) and forced vital capacity (FVC) (mean difference: 0.07 L, 95% CI: 0.02-0.12; I² = 78%), although these improvements approximate or fall below the established minimal clinically important difference for FEV1 (100 mL), suggesting modest clinical benefit at the population level. No statistically significant reduction in exacerbation risk was observed (risk ratio: 1.31, 95% CI: 0.73-2.36; I² = 76%); however, given the substantial heterogeneity and wide confidence interval, this finding should not be interpreted as evidence of no effect, particularly as individual trial data suggest exacerbation benefit may be concentrated in patients with more severe disease or frequent prior exacerbations. No significant differences were found for quality of life as measured by the St. George's Respiratory Questionnaire (mean difference: −6.26, 95% CI: −21.70 to 9.18; I² = 78%) or all-cause mortality (risk ratio: 1.02, 95% CI: 0.54-1.91; I² = 0%). Roflumilast produces statistically significant but modest improvements in lung function in patients with COPD; its effects on exacerbations, quality of life, and mortality remain uncertain, given substantial heterogeneity across studies. These findings support current guideline recommendations restricting roflumilast use to specific patient phenotypes, and further large-scale trials in more homogeneous populations are warranted to clarify its role in COPD management.

## Introduction and background

Chronic obstructive pulmonary disease (COPD) remains one of the leading causes of morbidity and mortality worldwide, characterized by persistent airflow limitation and chronic airway inflammation [1]. Acute exacerbations are a major driver of disease progression, hospitalization, and healthcare costs, with exacerbation frequency reported at 22%, 33%, and 47% in moderate, severe, and very severe COPD stages, respectively [2]. Reducing the frequency and severity of exacerbations is therefore a central goal of long-term COPD management [3].

Roflumilast, an oral phosphodiesterase-4 (PDE4) inhibitor, has emerged as a therapeutic option distinct from conventional bronchodilators and inhaled corticosteroids. It is designed as a nonsteroidal anti-inflammatory agent that targets both the systemic and pulmonary inflammation associated with COPD [4]. Unlike bronchodilators that act primarily on airway smooth muscle, roflumilast is thought to modulate the underlying inflammatory cascade, in which CD8+ T-lymphocytes, macrophages, and neutrophils play key pathogenic roles [5]. By inhibiting PDE4, roflumilast raises intracellular cyclic adenosine monophosphate in inflammatory and structural airway cells, a mechanism hypothesized to reduce neutrophilic airway inflammation and thereby improve lung function and lower exacerbation risk [6].

Since its approval, numerous randomized controlled trials (RCTs) have assessed roflumilast’s efficacy and safety in patients with stable, moderate-to-severe COPD, both as monotherapy and combined with long-acting bronchodilators or inhaled corticosteroid/long-acting β2-agonist regimens [7-9]. Findings across trials have been notably heterogeneous. Several large trials and pooled analyses report statistically significant, though modest, reductions in exacerbation rates and improvements in trough and post-bronchodilator forced expiratory volume in one second (FEV1) (weighted mean difference in exacerbation rate = −0.23, 95% CI: −0.33 to −0.13; improvement in trough FEV1 = 53.52 mL, 95% CI: 42.49-64.55) [10], whereas other syntheses have failed to demonstrate improvement in health-related quality of life on the St. George's Respiratory Questionnaire (SGRQ), or any reduction in mortality [11]. An earlier Cochrane-based analysis of nine RCTs (n = 9,211) similarly found significant gains in FEV1 and forced vital capacity (FVC) but only small absolute differences in patient-reported outcomes and exacerbation rates [12].

Given this heterogeneity in study populations, comparators, background therapies, and reported outcomes, an updated, comprehensive meta-analysis is warranted to clarify roflumilast’s overall efficacy and safety profile in COPD. This review aims to synthesize evidence from available studies to quantify roflumilast’s effects on exacerbation rate, lung function, and adverse events, informing its role in contemporary COPD management.

## Review

Methodology

This systematic review and meta-analysis was conducted in accordance with the Preferred Reporting Items for Systematic Reviews and Meta-Analyses (PRISMA) guidelines [13]. The protocol of this study was registered with the International Prospective Register of Systematic Reviews (PROSPERO).

Literature Search and Search Strategy

A comprehensive literature search was undertaken to identify studies evaluating the efficacy and safety of roflumilast in patients with COPD, following the PRISMA guidelines. Electronic searches were conducted in PubMed/MEDLINE, Embase, the Cochrane Central Register of Controlled Trials (CENTRAL), Web of Science, Scopus, and ClinicalTrials.gov from 1 January 2011 to 15 July 2026.

To maximize study identification, the reference lists of all included articles, relevant systematic reviews, and conference proceedings were manually examined for additional eligible studies not identified through the electronic database search. No language restrictions were imposed during the initial search, and non-English publications were translated whenever feasible. Literature searching and study screening were carried out independently by two reviewers, with any disagreements resolved by discussion or, when necessary, consultation with a third reviewer.

Search Strategy

The search strategy combined Medical Subject Headings (MeSH) terms and free-text keywords related to the intervention and condition of interest. Search terms included combinations of “roflumilast,” “Daliresp,” “phosphodiesterase-4 inhibitor,” “PDE4 inhibitor,” “chronic obstructive pulmonary disease,” and “COPD,” joined using the Boolean operators AND/OR. An example PubMed search string was: (“roflumilast”[MeSH] OR “roflumilast” OR “PDE4 inhibitor”) AND (“chronic obstructive pulmonary disease”[MeSH] OR “COPD” OR “chronic bronchitis”). The full search strategy was adapted appropriately for the syntax of each database. All identified records were imported into reference management software (EndNote, Clarivate, Philadelphia, PA) for duplicate removal, screening, and eligibility assessment.

Study Selection

Study selection was undertaken independently by two reviewers. Titles and abstracts retrieved from the literature search were initially screened against the predefined eligibility criteria to identify potentially relevant studies. The full texts of eligible articles were subsequently assessed independently by the same reviewers to determine final study inclusion. Any discrepancies arising during either stage of the selection process were resolved through discussion, with a third reviewer consulted when agreement could not be achieved. The entire selection process, including the numbers of records identified, screened, excluded (with reasons), and ultimately included, was documented in accordance with PRISMA recommendations and is presented in the PRISMA flow diagram.

Eligibility Criteria

Studies were eligible for inclusion if they were RCTs, quasi-experimental studies, or observational studies (cohort or case-control designs) evaluating roflumilast in adult patients with a confirmed clinical diagnosis of COPD, with or without chronic bronchitis, of any severity, while studies enrolling patients with a primary diagnosis of asthma, bronchiectasis, interstitial lung disease, or other overlap syndromes were excluded unless COPD-specific outcomes were reported separately; eligible studies had to evaluate roflumilast at any dose, administered alone or in combination with standard COPD therapies, compared with placebo, usual care, or an active comparator, and report at least one outcome of interest, including frequency or rate of COPD exacerbations, change in lung function (FEV1, FVC), health-related quality of life, all-cause mortality, or exacerbation. Case reports, case series, editorials, letters, conference abstracts without full data, animal or in vitro studies, narrative reviews, duplicate publications, and studies with overlapping patient populations (in which case the study with the longer follow-up or larger sample size was retained) were excluded from the final analysis.

Quality Assessment

The methodological quality of included studies was independently assessed by two reviewers, with disagreements resolved through discussion or consultation with a third reviewer, using tools appropriate to each study design. For RCTs, risk of bias was evaluated using the Cochrane Risk of Bias tool, version 2 (RoB 2), which assesses bias arising from the randomization process, deviations from intended interventions, missing outcome data, measurement of the outcome, and selection of the reported result; each domain was rated as “low risk,” “some concerns,” or “high risk,” and an overall risk-of-bias judgment was derived accordingly [14].

For observational studies (cohort and case-control designs), the Newcastle-Ottawa Scale (NOS) was applied, evaluating study quality across three domains, i.e., selection of study groups, comparability of groups, and ascertainment of the outcome or exposure of interest, with studies scoring a maximum of nine stars, and those scoring ≥7, 5-6, and ≤4 stars categorized as high, moderate, and low quality, respectively [15]. The full quality-assessment ratings for each included study are presented in the accompanying Data Extraction and Quality Assessment sections (RoB 2 and NOS tables).

Data Extraction

Data extraction was performed independently by two reviewers using a standardized, pilot-tested extraction form. Any disagreements were resolved through discussion, with consultation from a third reviewer when consensus could not be reached. Information collected from each eligible study included study identification, methodological characteristics, participant demographics, intervention and comparator details, and reported outcome measures. Key study characteristics, including first author, publication year, study design, geographical region, sample size, roflumilast dose, follow-up duration, mean participant age, and the number of male participants, are summarized in the Data Extraction table.

Data Analysis

All statistical analyses were conducted using Review Manager (RevMan) version 5.4 (The Cochrane Collaboration, London, UK). Continuous outcomes, including FEV1, FVC, and SGRQ scores, were pooled using mean differences (MDs) with corresponding 95% confidence intervals (CIs). Dichotomous outcomes, including COPD exacerbations and all-cause mortality, were summarized as risk ratios (RRs) with 95% CIs.

A random-effects model was prespecified for all meta-analyses to account for the expected clinical and methodological variability among the included studies, including differences in study design, patient characteristics, background treatments, roflumilast dosage, and duration of follow-up. For studies reporting outcomes at multiple follow-up time points, data from the longest available follow-up were selected for the primary analysis to ensure consistency across studies.

Between-study heterogeneity was evaluated using the Cochrane Q (chi-square) test and quantified with the I² statistic. Heterogeneity was interpreted according to conventional thresholds, with I² values of 25%, 50%, and 75% indicating low, moderate, and high heterogeneity, respectively.

Results

**Figure 1:**
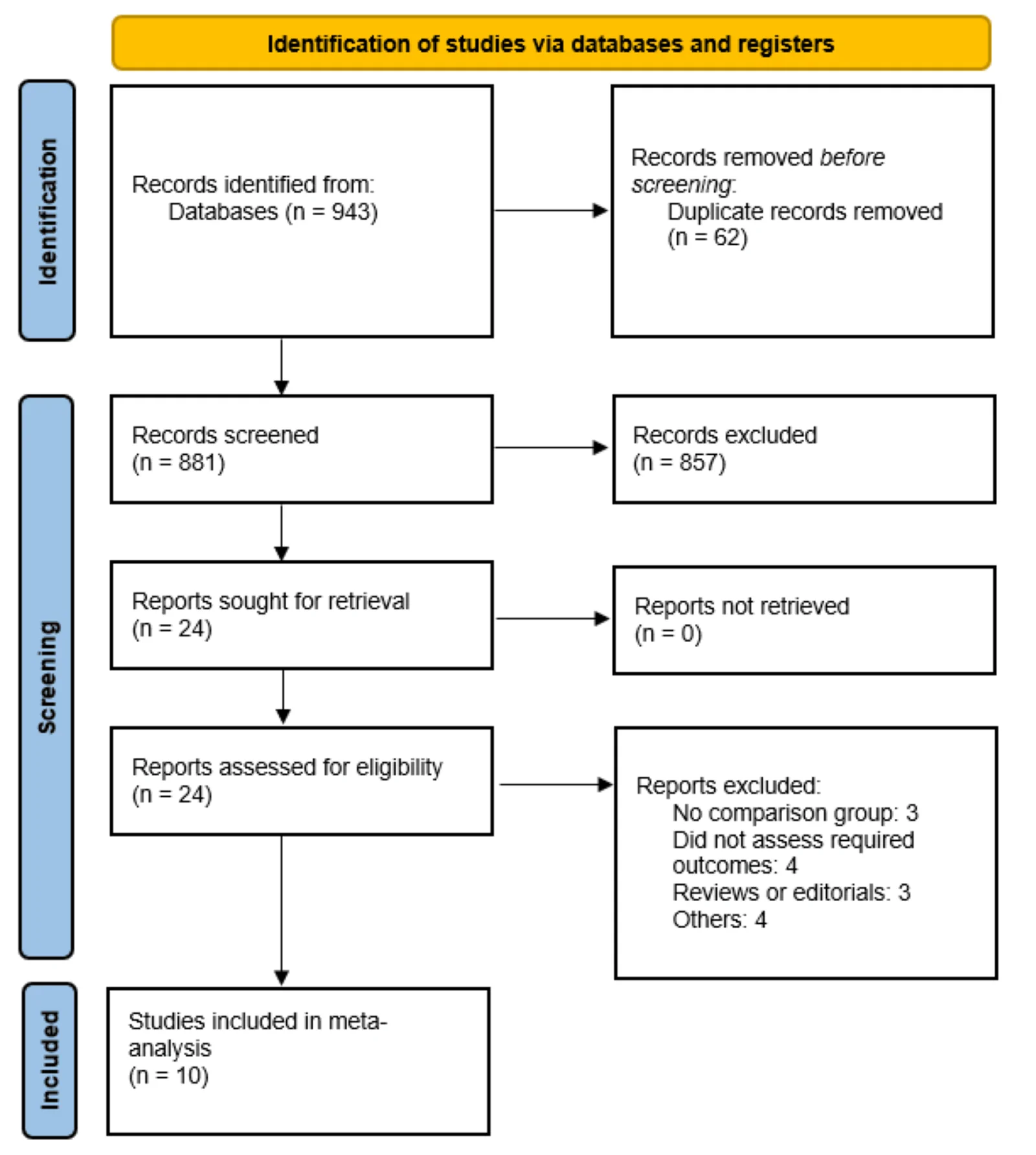
Study selection process (PRISMA).

Figure 1 illustrates the PRISMA flow diagram summarizing the study selection process. The initial database search identified 943 records. After duplicate removal, 881 articles underwent title and abstract screening. Subsequently, the full texts of 24 potentially eligible studies were retrieved and assessed against the predefined inclusion and exclusion criteria. Ultimately, 10 studies met the eligibility criteria and were included in the meta-analysis. The characteristics of the included studies are summarized in Table 1, with the majority being RCTs. The methodological quality and risk of bias assessments of the included studies are presented in Table 2.

**Table 1:** Characteristics of included studies. RCT: randomized controlled trial; NR: not reported; KOCOSS: Korea Chronic Obstructive Pulmonary Disorders Subgroup Study; µg: microgram.

Author	Year	Study design	Region/Country	Sample size (n)	Dose	Follow-up	Mean age (years)	Males, n (%)
De Backer et al. [16]	2014	RCT	NR	1173 (567 roflumilast and 606 control)	500 µg once daily	NR	Control: 64; roflumilast: 64	Control: 400; roflumilast: 387
Laique et al. [17]	2026	Quasi-experimental (open-label)	Pakistan	80 (40 roflumilast and 40 control)	500 µg once daily	6 months	Control: 63.80; roflumilast: 67.80	Control: 18; roflumilast: 25
Lee et al. [18]	2011	RCT	Asia-Pacific (Hong Kong, Malaysia, Philippines, South Korea, Taiwan)	410 (203 roflumilast vs. 207 control)	500 µg once daily	3 months	Control: 67; roflumilast: 68	Roflumilast: 188; control: 195
Lee et al. [19]	2025	Retrospective cohort study (KOCOSS registry)	South Korea	180 (18 roflumilast vs. 162 no roflumilast), all with radiologically confirmed bronchiectasis	Real-world prescribed dosing (not protocol-fixed)	36 months	Roflumilast: 71.4; control: 68.8	Roflumilast: 18; control: 138
Liu et al. [20]	2018	RCT	China	120 (60 roflumilast vs. 60 control)	500 µg once daily	12 months	Roflumilast: 64.4; control: 65.1	Roflumilast: 45; control: 42
Mackay et al. [21]	2017	RCT	United Kingdom	81 (38 roflumilast vs. 43 control)	500 µg once daily	2 months	Roflumilast: 72.9; control: 70.5	Roflumilast: 22; control: 28
Martinez et al. [9]	2015	RCT	21 countries (multinational)	1,935 (969 roflumilast vs. 966 control)	500 µg once daily	52 weeks	Roflumilast: 65; control: 65	Roflumilast: 718; control: 725
Martinez et al. [22]	2016	RCT	Multinational (US and non-US centers)	2,352 (1,178 roflumilast vs. 1,174 control)	500 µg once daily	52 weeks	Roflumilast: 64.4; control: 64.5	Roflumilast: 821; control: 794
O'Donnell et al. [23]	2012	RCT	Canada, France, Germany, Hungary	250 (127 roflumilast vs. 123 control)	500 µg once daily	12 weeks	Roflumilast: 60; control: 59	Roflumilast: 93; control: 103
Rennard et al. [6]	2011	RCT	Multinational	2,686 (1,327 roflumilast vs. 1,359 control)	500 µg once daily	52 weeks	Roflumilast: 64.7; control: 64.4	Roflumilast: 958; control: 974

**Table 2:** Quality assessment of included studies.

Study	D1: Randomization process	D2: Deviations from intended interventions	D3: Missing outcome data	D4: Measurement of the outcome	D5: Selection of reported result	Overall risk of bias
De Backer et al. (2014) [16]	Low risk	Low risk	Some concerns	Low risk	Low risk	Low risk
Laique et al. (2026) [17]	High risk	Some concerns	Low risk	Some concerns	Low risk	High risk
Lee et al. (2011) [18]	Low risk	Low risk	Some concerns	Low risk	Low risk	Low risk
Liu et al. (2018) [20]	Low risk	Low risk	Low risk	Low risk	Low risk	Low risk
Mackay et al. (2017) [21]	Low risk	Low risk	Some concerns	Low risk	Low risk	Some concerns
Martinez et al. (2015) [9]	Low risk	Low risk	Some concerns	Low risk	Low risk	Some concerns
Martinez et al. (2016) [22]	Low risk	Low risk	Some concerns	Low risk	Low risk	Some concerns
O'Donnell et al. (2012) [23]	Low risk	Low risk	Low risk	Low risk	Low risk	Low risk
Rennard et al. (2011) [6]	Low risk	Low risk	Some concerns	Low risk	High risk	High risk
Quality assessment of observational studies						
Study	Selection	Comparison	Outcome assessment	Overall		
Lee et al. (2025) [19]	3	2	3	Good		

Meta-Analysis of Outcomes

**Figure 2:**
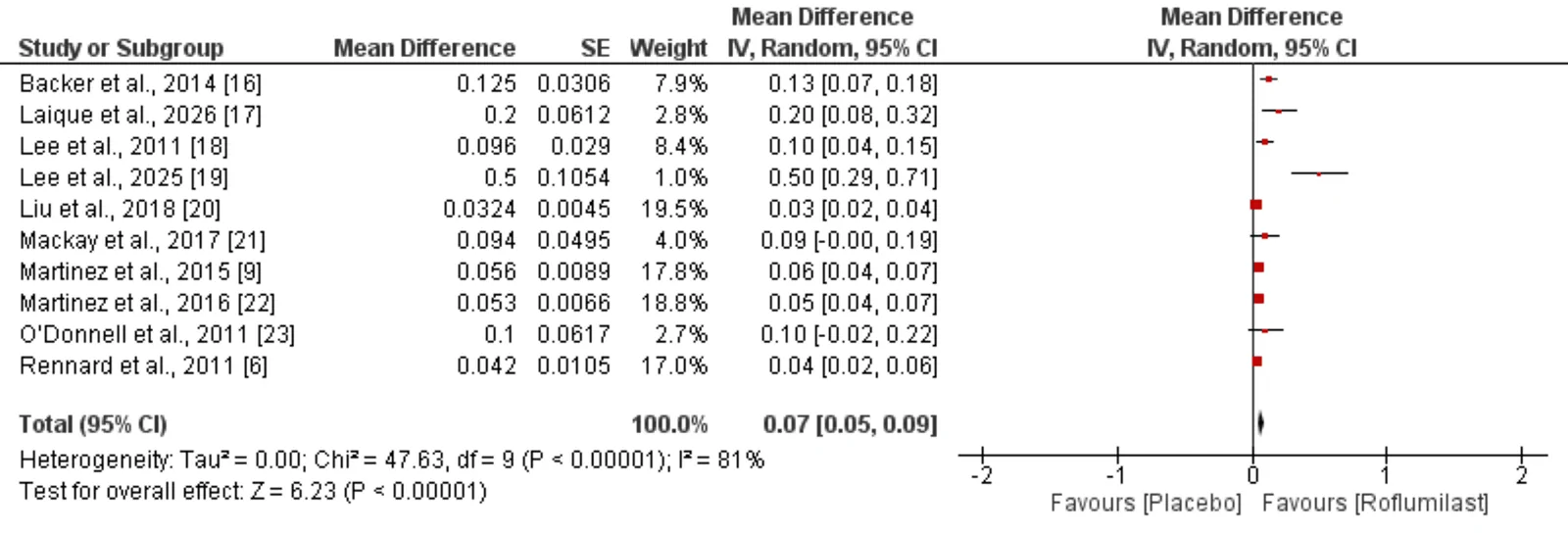
Comparison of forced expiratory volume between the two groups. References [6,9,16-23].

FEV1: Ten studies reporting on FEV1 were pooled in the meta-analysis, and the results are shown in Figure 2. Roflumilast was associated with a statistically significant improvement in FEV1 compared with placebo, with a pooled mean difference of 0.07 L (95% CI: 0.05 to 0.09) under a random-effects model. All included studies favored roflumilast over placebo. However, substantial statistical heterogeneity was observed across studies (I² = 81%), indicating considerable variability in effect size that cannot be attributed to chance alone. Sensitivity analysis was performed by removing observational studies, and findings of pooled RCTs showed similar results as pooled analysis of all studies (MD: 0.06, 95% CI: 0.04 to 0.08). Heterogeneity reduced from 81% to 72%.

**Figure 3:**
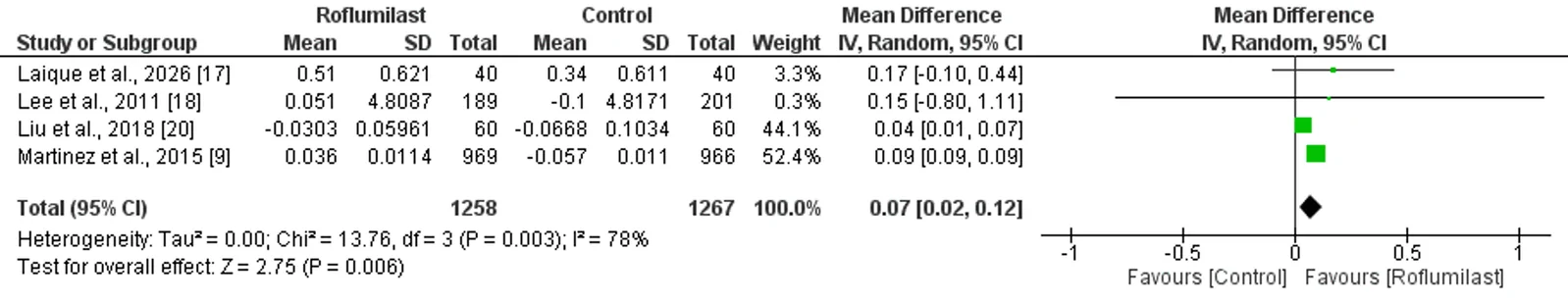
Comparison of forced vital capacity between the two groups. References [9,17,18,20].

FVC: Four studies reporting on FVC were pooled, comprising 1,258 patients in the intervention arm and 1,267 patients in the control arm, and the results are shown in Figure 3. Roflumilast was associated with a statistically significant improvement in FVC compared with control, with a pooled mean difference of 0.07 (95% CI: 0.02 to 0.12) under a random-effects model. All four studies numerically favored the intervention group. Substantial statistical heterogeneity was observed (I² = 78%), indicating that pooled results should be interpreted with caution.

**Figure 4:**
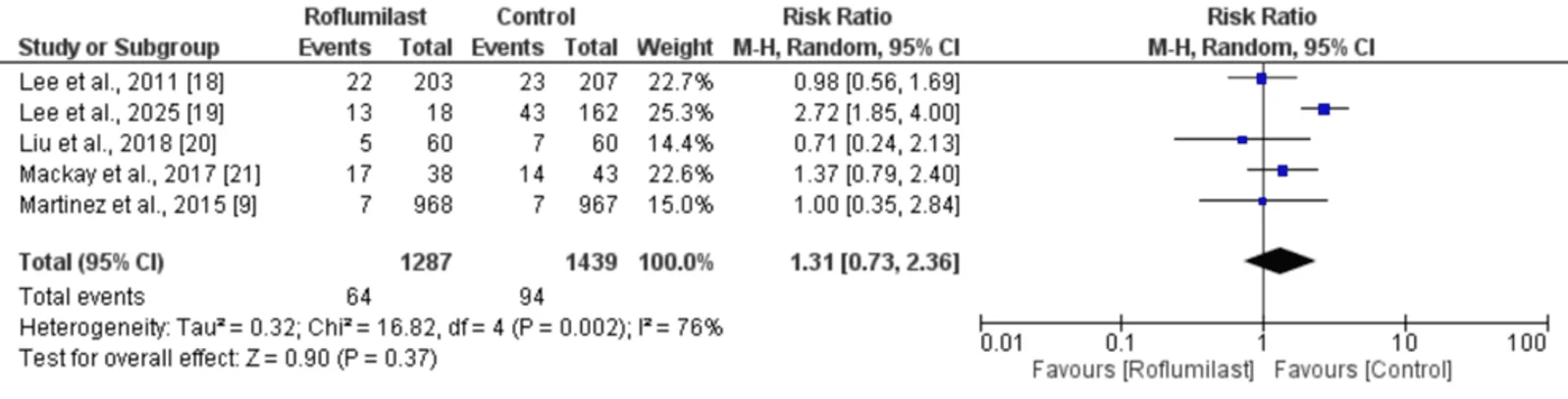
Comparison of exacerbations between the two groups. References [9,18-21].

Exacerbations: Five studies reported data on COPD exacerbations, comprising 1,287 patients in the experimental group and 1,439 patients in the control group, and the results are shown in Figure 4. Pooled analysis using a random-effects model showed no statistically significant difference in exacerbation risk between roflumilast and control, with a risk ratio of 1.31 (95% CI: 0.73 to 2.36). A total of 64 exacerbation events occurred in the experimental group compared with 94 in the control group. Moderate-to-substantial statistical heterogeneity was observed across studies (I² = 76%), indicating meaningful variability in effect estimates that limits confidence in the pooled null result. Sensitivity analysis was performed excluding the study conducted by Lee et al. (2025) [19]. Sensitivity analysis showed no significant difference between the two groups (RR: 1.08, 95% CI: 0.76 to 1.53). However, heterogeneity reduced from 76% to 0%.

Other outcomes: For health-related quality of life, assessed using the SGRQ, pooled analysis showed no statistically significant difference between roflumilast and control, with a mean difference of −6.26 (95% CI: −21.70 to 9.18). Although the point estimate numerically favored roflumilast, the wide confidence interval crossing the null, combined with substantial statistical heterogeneity (I² = 78%), precludes any firm conclusion regarding the impact of roflumilast on quality of life. For all-cause mortality, pooled analysis showed no statistically significant difference between roflumilast and control, with a risk ratio of 1.02 (95% CI: 0.54 to 1.91) and no observed statistical heterogeneity (I² = 0%), indicating consistent effect estimates across the included studies, as shown in Table 3.

**Table 3:** Secondary outcomes. SGRQ: St. George's Respiratory Questionnaire; MD: mean difference; CI: confidence interval. * Presented as a risk ratio with 95% CI.

Outcome	MD (95% CI)	I-square
SGRQ	-6.26 (-21.70 to 9.18)	78%
Mortality	1.02 (0.54 to 1.91)*	0%

Discussion

This study combined evidence from 10 studies, including RCTs, a quasi-experimental study, and observational cohort studies, to examine the efficacy of roflumilast in patients with COPD. The pooled analysis demonstrated that roflumilast significantly improved pulmonary function, as reflected by increases in FEV1 and FVC. However, no statistically significant benefits were observed for exacerbations, health-related quality of life measured using SGRQ, or all-cause mortality. Overall, these findings both support and extend the current understanding of roflumilast's therapeutic role in COPD.

COPD exacerbations are acute episodes characterized by worsening respiratory symptoms that often require intensification of medical therapy and, in severe cases, hospitalization [24]. Consequently, contemporary COPD management aims not only to improve symptoms but also to minimize exacerbations, delay disease progression, and reduce mortality [25]. Roflumilast, a selective PDE4 inhibitor, exerts anti-inflammatory effects by reducing inflammatory activity within the airways. For this reason, it has been approved as an add-on maintenance therapy in combination with inhaled corticosteroids (ICS) and long-acting bronchodilators for patients with severe COPD who remain at increased risk of recurrent exacerbations [26].

Our pooled results indicate that roflumilast significantly enhances lung function, particularly FEV1, compared with placebo. Improvement in FEV1 is widely regarded as a key indicator of treatment efficacy in COPD and remains the cornerstone for disease diagnosis, severity classification, and therapeutic evaluation [25,27]. Although Mackay et al. [21] reported numerically greater improvements in FEV1 that did not achieve statistical significance, Watz et al. [28] demonstrated clinically meaningful improvements across all treatment groups regardless of dosage or treatment duration. Taken together, these findings consistently suggest that roflumilast produces favorable effects on pulmonary function across diverse clinical settings.

Several previous meta-analyses have also assessed the efficacy of roflumilast in COPD, and our findings both confirm and expand upon the existing literature. Yan et al. [11], who synthesized data from multiple RCTs involving patients with stable COPD, reported a significant reduction in exacerbation rates (weighted mean difference: −0.23, 95% CI: −0.33 to −0.13) together with an improvement in trough FEV1 of 53.52 mL (95% CI: 42.49-64.55). While our analysis similarly demonstrates beneficial effects on lung function, we did not observe a statistically significant reduction in exacerbation risk. This discrepancy may reflect differences in study populations, disease characteristics, treatment settings, follow-up periods, and the inclusion of observational evidence in the present review.

An important strength of the current review is the inclusion of evidence evaluating roflumilast beyond stable COPD. Unlike previous meta-analyses [10-12], which primarily focused on maintenance therapy in stable disease, our review incorporated the TREAT trial by Mackay et al. [21], thereby capturing data from patients experiencing acute exacerbations. The inclusion of this study provides additional insight into the clinical context in which roflumilast may be most effective. Collectively, the available evidence suggests that the anti-inflammatory effects of roflumilast are likely to translate into greater clinical benefit during stable maintenance treatment than during acute exacerbations or in patients with more complex disease phenotypes, such as COPD with coexisting bronchiectasis, as reported by Lee et al. [19]. These observations have important implications for selecting appropriate patients for therapy in routine clinical practice.

Our findings should be interpreted in the context of current treatment guidance. The 2026 GOLD report recommends roflumilast specifically for patients with FEV1 <50% predicted, chronic bronchitis, and a history of exacerbations requiring hospitalization, positioning it as an add-on therapy for patients with persistent exacerbations despite optimized inhaled treatment, rather than as a first-line or broad-population intervention. This targeted positioning is consistent with the pattern observed in our review: while roflumilast produced consistent improvements in lung function across the general COPD population studied, its exacerbation benefit was most apparent in patients with more severe disease and a higher exacerbation burden. This is illustrated by Martinez et al. [22], in which the overall RE²SPOND population did not show a statistically significant reduction in exacerbations, but a post hoc analysis restricted to patients with more than three exacerbations in the prior year demonstrated a 39% reduction (RR: 0.61, 95% CI: 0.39-0.95), and by Martinez et al. [9] (REACT), which similarly demonstrated significant benefit in a population selected for frequent exacerbations and chronic bronchitis. These findings support the GOLD recommendation that roflumilast is best reserved for a specific phenotype, including patients with severe airflow limitation, chronic bronchitis, and frequent or hospitalization-associated exacerbations, rather than applied broadly across the COPD population.

The clinical relevance of the observed lung function improvements also warrants consideration alongside their statistical significance. The minimal clinically important difference (MCID) for trough FEV1 in COPD is widely accepted as approximately 100 mL, representing the smallest change patients are able to perceive as beneficial [29]. Our corrected pooled FEV1 improvement therefore falls below this threshold, suggesting that while roflumilast produces a statistically robust and biologically plausible improvement in lung function, its magnitude may be of limited or borderline clinical significance for the average patient in this population. This distinction is important: individual trials included in our analysis reported FEV1 improvements ranging from approximately 30 mL [20] to 94 mL [21], indicating that clinically meaningful benefit, where present, is likely concentrated in specific patient subgroups such as those with more severe airflow limitation or frequent exacerbations, consistent with the GOLD-recommended patient profile discussed above rather than reflecting a uniform effect across the general COPD population. No validated MCID currently exists for FVC in the general COPD population, which limits similar interpretation for this outcome, although values reported in the literature (139-379 mL depending on disease severity and population) suggest our pooled FVC improvement is also modest in absolute terms.

Limitations

The present review has several notable strengths. By incorporating evidence from both controlled clinical trials and real-world observational studies, it provides a broader evaluation of roflumilast's effectiveness in routine clinical practice. Nevertheless, certain limitations should be acknowledged. The inclusion of studies with different methodological designs, patient populations, treatment protocols, and follow-up durations introduced substantial clinical and methodological heterogeneity, particularly in the analyses of exacerbation outcomes. Such heterogeneity may have reduced the precision of the pooled estimates and limited the generalizability of the findings. Furthermore, because the analysis relied exclusively on published aggregate data, subgroup analyses to identify patient populations deriving the greatest benefit from roflumilast were not feasible. Future studies with larger sample sizes and individual patient-level data are required to address this limitation.

Despite these constraints, the present findings identify several priorities for future research. Additional studies should evaluate clinically relevant outcomes beyond lung function, including exercise tolerance, duration of hospitalization, and biochemical markers such as inflammatory mediators and cytokines, to further clarify the mechanisms and clinical benefits of roflumilast in COPD management [30]. Further investigation is also warranted to determine optimal combination treatment strategies and better understand potential interactions between roflumilast and other COPD medications. Given the increasing emphasis on medication safety [31], long-term studies examining survival, adverse events, and cost-effectiveness are also needed. Finally, further research should continue to evaluate the long-term efficacy and safety of roflumilast across different stages of COPD, including patients with milder disease who remain underrepresented in the current evidence. We also need future studies to analyze groups that are more likely to benefit from this therapy.

## Conclusions

Roflumilast consistently improves lung function in patients with COPD; however, given the substantial heterogeneity observed and the wide confidence interval for the pooled exacerbation estimate, our findings should not be interpreted as establishing an absence of benefit on exacerbation risk. Rather, the non-significant pooled result likely reflects clinical and methodological variability across included studies, including differences in population severity, exacerbation history, and study design. Notably, evidence from individual large-scale trials suggests that exacerbation benefit may be concentrated in specific patient subgroups, such as those with frequent prior exacerbations or hospitalization history, consistent with current GOLD-recommended patient selection criteria. Substantial heterogeneity across studies limits the certainty of these findings, and further large-scale trials with more homogeneous populations are warranted to clarify roflumilast's role in reducing exacerbations in COPD.
